# Investigating the Influence of Anthropogenic Forcing on Observed Mean and Extreme Sea Level Pressure Trends over the Mediterranean Region

**DOI:** 10.1100/2012/525303

**Published:** 2012-05-03

**Authors:** Armineh Barkhordarian

**Affiliations:** Helmholtz-Zentrum Geesthacht Centre for Materials and Coastal Research, Max-Planck-Straße, 21502 Geesthacht, Germany

## Abstract

We investigate whether the observed mean sea level pressure (SLP) trends over the Mediterranean region in the period from 1975 to 2004 are significantly consistent with what 17 models projected as response of SLP to anthropogenic forcing (greenhouse gases and sulphate aerosols, GS). Obtained results indicate that the observed trends in mean SLP cannot be explained by natural (internal) variability. Externally forced changes are detectable in all seasons, except spring. The large-scale component (spatial mean) of the GS signal is detectable in all the 17 models in winter and in 12 of the 17 models in summer. However, the small-scale component (spatial anomalies about the spatial mean) of GS signal is only detectable in winter within 11 of the 17 models. We also show that GS signal has a detectable influence on observed decreasing (increasing) tendency in the frequencies of extremely low (high) SLP days in winter and that these changes cannot be explained by internal climate variability. While the detection of GS forcing is robust in winter and summer, there are striking inconsistencies in autumn, where analysis points to the presence of an external forcing, which is not GS forcing.

## 1. Introduction

Applying formal detection methods, observed patterns of global December-to-February sea level pressure changes have been shown to be inconsistent with simulated internal variability but consistent, after rescaling its amplitude, with simulations driven by anthropogenic forcings. Therefore the SLP response to external forcing is considered to have been detected [1, 2]. However, the trend of SLP in the North Hemisphere has generally been found to be larger than that simulated trend in response to natural and anthropogenic influence [2]. Greenhouse gases, stratospheric ozone depletion, volcanic aerosol, and solar forcing have all been cited as having an impact on SLP [1, 3, 4].

Wang et al. [5] attempted to detect external influence on global SLP trends in each of the four seasons, but did not find detectable external influence on SLP in any season. Gillett and Stott [6] applied for the first time a formal attribution analysis and demonstrated that externally forced SLP trends are observed in all four seasons, with simulated and observed decreases in SLP at high latitudes and increases elsewhere. They found that the observed pattern of seasonal and zonal mean SLP changes is inconsistent with simulated internal variability and that the anthropogenic influence on SLP is therefore detectable, regardless of other natural influences. But when they separately considered the mid- and high-latitude regions of both hemispheres and the tropics and subtropics, they found that the external influence is only detectable in the low-latitude region, where models and observations show positive trends in SLP and where internal variability is low. In contrast, the anthropogenic influence could not be detected at mid- and high-latitude regions of either hemisphere.

Here, we apply a different approach to investigate whether greenhouse gases and tropospheric sulphate aerosols (GS)—the main human influences on climate—had a detectable influence on recently observed SLP changes over the Mediterranean region. This method has been earlier applied to near-surface temperature [7] and precipitation [8]. The results obtained indicate that most likely (with less than 2.5% risk) GS forcing is a plausible explanation for the observed warming in this region and that present trends may be understood of what will come more so in the future, allowing for a better communication of the societal challenges to meet in the future [7]. However, in terms of precipitation, the expectation of future precipitation change over the Mediterranean is different from what we observe. Obtained results show that factors other than GS-forcing may have played a significant role in shaping the precipitation trends during the recent decades in this region. Therefore, recent observed changes cannot be used to illustrate the future expected changes of precipitation over the Mediterranean region [8].

In the present study we analyze mean sea level pressure (SLP) trends and investigate whether the observed changes are consistent with natural (internal) variability, and if not, whether they are consistent with what models simulate as response of SLP to GS forcing (greenhouse gases and sulphate aerosols, GS). Therefore we compare trends in observed SLP over the period from 1975 to 2004 with the response to GS forcing derived from the set of global climate model simulations provided through the World Climate Research Programmes (CMIP3, [9]).

Extremes of climate events are increasingly being recognized as key aspects of climate change. In view of this, we further investigate the role of GS forcing in the frequencies of extreme low and high mean SLP days. We used the *P* ^10^ and the *P* ^90^ percentiles of the distribution of daily SLP as a threshold to define the extreme daily values. Days with SLP less than the 10th percentile value are defined as low SLP days, and those greater than the 90th percentile are defined as high SLP days. We thus obtain the series of seasonal frequencies of the days with extreme SLP. This should allow it to be decided whether the temporal trend of the mean values of sea level pressure is due to the behaviour of its extreme values or not [10, 11].

For the first time this method is being applied to Mediterranean Sea level pressure. In contrast to other studies that have often focused on the winter season, we analyze the different seasons separately. The method used in this work allows us to deal with each model separately and present results for the 17 models individually.

The remainder of this paper is structured as follows. Details on the observational and model data are given in Section 2. The methodology, including estimating anthropogenic climate change signal, estimating natural (internal) variability, comparing the patterns of changes, and the method used to obtain the significance of pattern similarity statistics is presented in Section 3. The results including the detection of external forcing and the consistency with GS forcing at spatial mean level and at pattern level are shown in Section 4. The robustness of the results against observationally based internal variability is shown in Section 5. The fingerprint of NAO is discussed in Section 6. The results of analysis about the frequency of extreme SLP days are presented in Section 7. The summary and conclusions are in Section 8. 

## 2. Observations and Model Data

 In this study the Mediterranean area is defined as the region from 25°N to 50°N and 10°W to 40°E. We use the HadSLP2.0 noninterpolated gridded monthly sea level pressure observations for the period 1850–2004 [12]. We use global simulations with 17 coupled atmosphere-ocean general circulation models (AOGCMs) to estimate the response of of mean SLP to anthropogenic GS forcing.The simulations are included in the World Climate Research Programme's (WCRP's) Coupled Model Intercomparison Project (CMIP3, [9]). The simulations are driven by the IPCC SRES A1B scenario, ending with a CO2 concentration of 720 ppm by the year 2100. The name of the models and the number of runs with each individual model are given in Table 1.

For changes in the frequency of extreme daily SLP, consistency analysis is based on the daily mean sea level pressure gridded reconstructions, EMSLP [13], available for the period 1850–2003. The daily SLP data in the CMIP3 archive is not completely available, and thus, to estimate the anthropogenic climate change signal of daily SLP extremes, we only use the INGV model [14], which is a global coupled ocean-atmosphere general circulation model (AOGCM), coupled with a high-resolution (7 Km) regional model of the Mediterranean Sea. 

## 3. Methodology

 Our analyses are based on the approach used in ([7] see also [15, 16]). In the first step, we investigate whether the externally forced, changes are detectable in the observed trends; therefore annual and seasonal observed mean SLP trends are compared with an estimation of the natural (internal) variability of the SLP trends (Section 3.3). If externally forced changes are detectable, in the second step we assess whether the observed trends are consistent with what models simulate as response of SLP to anthropogenic forcing (greenhouse gases and sulphate aerosols, GS). For this purpose, we compare trends in observed SLP over the period 1975–2004 with the anthropogenic (GS) climate change signal derived from a set of climate model simulations (Section 3.1). The comparison is carried out using several pattern-similarity statistics based on regression analysis and outlined in Section 3.2. Consistency of observed trend patterns with GS signal pattern is claimed in cases where the uncertainty range of regression indices, derived from control runs, does not include zero but includes unit scaling.

### 3.1. Anthropogenic Climate Change Signal Estimates

 In this study two methods are used to estimate anthropogenic (GS) signal. On the one hand, we use time-slice experiment and define the anthropogenic climate change signal as the difference between the last decades of the 21st century (2071–2100) and the reference climatology (1961–1990). We assume a linear development from 1961–2100, and the resulting signal is scaled to mileibars of SLP change per year. Using well-separated time slices, 110 years in this study, has the advantage of increasing the signal-to-noise ratio and avoiding the need to average multiple models to get good signal estimates. Therefore, this method allows us to use different climate models and explicitly deal with individual models separately. A list of the climate models and the number of ensemble members of the individual models used in this study is given in Table 1.

On the other hand, we estimate the anthropogenic signal from model simulations, forced with estimates of historical anthropogenic forcing only (ANT), including greenhouse gases and sulphate aerosols (GS). The models used are BCCR-BCM2.0 (1 run), CNRM-CM3 (1 run), CSIRO-MK3.0 (1 run), GISS-AOM (2 runs), INGV-ECHAM4 (1 run), ECHAM5/MPI-OM (4 runs), CCCMA-CGCM3-1 (5 runs), CCCMA-CGCM3-T63 (1 run), and UKMO-HadGEM1 (1 run). Since the 20th century simulations generally finished in 2000, it was necessary to use outputs from SRES A1B scenario for the last 4 years of the simulation period. The multimodel ensemble mean is used here since it provides better signal estimate [17]. This averaging removes some traces of internal variability in the GS signal pattern. Since individual realizations can be regarded as being statistically independent, the uncertainty from internal variability in GS patterns decreases as *n* ^−0.5^ where *n* is the ensemble size [18]. Therefore, by using ensemble mean of 17 simulations in this study, the internal variability decreases and leads to increasing the signal-to-noise ratio by a factor of 4 in estimated anthropogenic patterns. This method is mainly used in this study to evaluate the robustness of the obtained results.

### 3.2. Comparing the Patterns of Change

 The comparison of observed and anthropogenic climate change signal patterns is carried out using four pattern similarity statistics. We examine the similarity between the two patterns by using uncentered and centred pattern correlation statistics (1) and(2)

(1) $$\begin{array}{cc} UC\left(O,P\right) & =\frac{\sum_{i=1}^{n}P_{i}\cdot O_{i}}{\sqrt{\sum_{i=1}^{n}P_{i}^{2}\cdot \sum_{i=1}^{n}O_{i}^{2}}}, \end{array}$$

(2) $$\begin{array}{cc} CC\left(O,P\right) & =\frac{\sum_{i=1}^{n}\left(P_{i}-\overset{®}{P}\right)\cdot \left(O_{i}-\overset{®}{O}\right)}{\sqrt{\sum_{i=1}^{n}{\left(P_{i}-\overset{®}{P}\right)}^{2}\cdot \sum_{i=1}^{n}{\left(O_{i}-\overset{®}{O}\right)}^{2}}}. \end{array}$$

The index subscript *i* = 1,…, *n* counts the spatial points. *O*_*i*_ and *P*_*i*_ refer to the observed and expected pattern of change, respectively.We also use uncentred and centred regression indices (3) and (4), which unlike the correlation statistics, also include information about the relative magnitude of the observed and model projected trend patterns. Uncentred statistics have the advantage of including information about the area mean change, while centred statistics focus on the pattern of spatial anomalies about the mean [19, 20]

(3) $$\begin{array}{cc} UR\left(O,P\right) & =\frac{\sum_{i=1}^{n}P_{i}\cdot O_{i}}{\sum_{i=1}^{n}P_{i}^{2}}, \end{array}$$

(4) $$\begin{array}{cc} R\left(O,P\right) & =\frac{\sum_{i=1}^{n}\left(P_{i}-\overset{®}{P}\right)\cdot \left(O_{i}-\overset{®}{O}\right)}{\sum_{i=1}^{n}{\left(P_{i}-\overset{®}{P}\right)}^{2}}. \end{array}$$

In order to have a measure without the effect of spatial pattern information, we also compare the area-mean changes of observed and anthropogenic signal patterns. Trends in observations have been calculated using ordinary least squares linear regression.

### 3.3. Estimating Natural (Internal) Variability

 We estimate natural (internal) variability from control integrations of CMIP3 climate models, which are preindustrial control experiments with all forcings held constant. A list of the climate models and the number of years used from control integrations to estimate the natural (internal) variability is given in Table 1. From 6,200-year control runs we draw 206 nonoverlapping 30-year segments to estimate chaotic variability from weather and other sources that would be present irrespective of any external influence on the climate system [18].

The quantiles of the pattern similarity indices of control run segments on the GS signal patterns are then used to test the null hypotheses that the pattern similarity statistics obtained between observation and modelled SLP response to GS arise by chance. We further test whether the similarity statistics are compatible with unit scaling, indicating consistency with GS forcing.

## 4. Results

**Figure 1:**
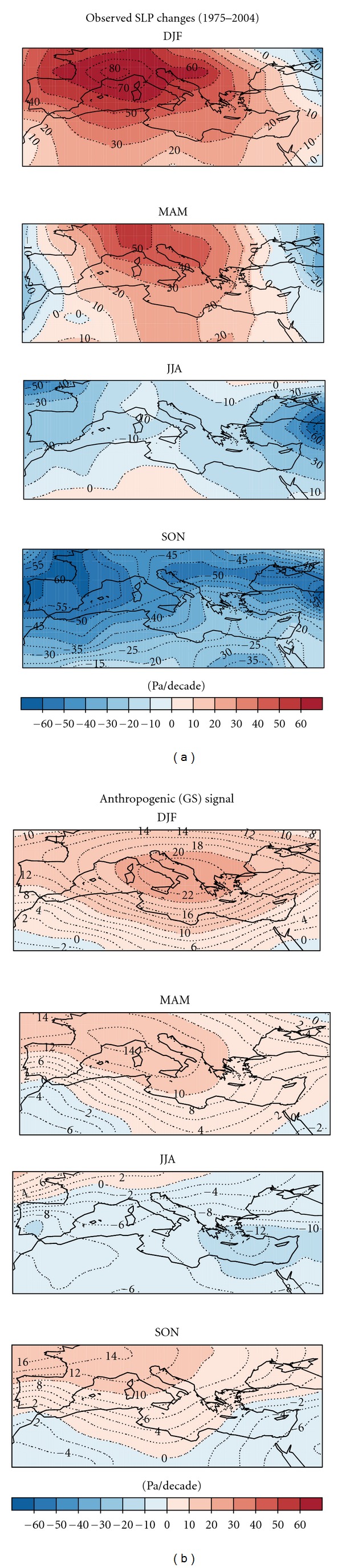
(a) Observed pattern on SLP derived from HadSLP2.0 dataset (1975–2004). (b) Projected GS signal pattern estimated from two well-separated time slices (2071–2100 minus 1961–90 mean scaled to change per decade) ensemble mean of 17 models from CMIP3 archive.

**Figure 2:**
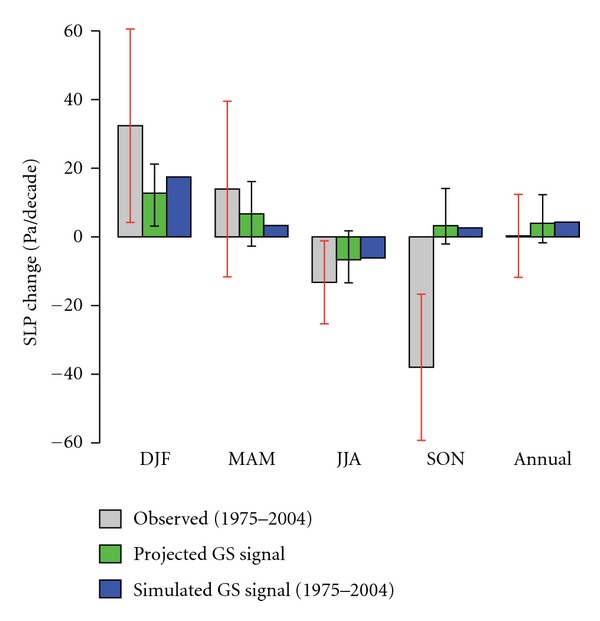
Observed seasonal and annual area mean changes of mean sea level pressure over the period 1975–2004 (grey bars) in comparison with the projected GS signals (GS) estimated from two well-separated time slices, according to SRES A1B scenario using 17 models (green bars) and the simulated GS signal based on ensemble mean of 17 ANT simulations conducted with 9 models forced with estimated historical GS forcing only (blue bars). The black whiskers indicate the spread of trends of 17 climate change projections used in this study. The red whiskers indicate the 90% uncertainty range of observed trends, derived from control variability (206 nonoverlapping 30-year segments derived from 6,200-year control runs).

Figure 1 displays the observed pattern of change of mean sea level pressure (SLP) during the period 1975–2004 in comparison with the projected GS signal pattern (ensemble mean of 17 models) based on the time-slice experiments, and Figure 2 compares the observed and projected (simulated) area-mean changes of SLP. In winter (DJF) the observed trends show an area of increased SLP centred over the Mediterranean, which extends to the north in spring (MAM).This area of high pressure is reproduced in response of SLP to GS forcing in all 17 models and, thus also in the multimodel mean. However, the climate models underestimate the magnitude of the SLP response in winter (see Figure 2). In summer (JJA) the observed trend pattern is characterized by negative trends, which is in agreement with response of SLP to GS forcing (Figures 1 and 2).

In autumn (SON), models suggest that anthropogenic forcing should have caused a small increase in mean SLP. However, the observed trend shows areas of pronounced decrease in mean SLP over the east and west of the Mediterranean (Figures 1 and 2), which contradicts 16 of the 17 climate change projections. As a consequence of these low-pressure areas, the Mediterranean region has unexpectedly experienced an upward trend in the amount of precipitation over the last few decades, which is strongly at odds with dry and stable conditions projected by climate change scenarios [8].

Figure 2 also displays the comparison between projected GS signal (derived from two well-separated time slices, 17 climate change projections) and simulated GS signal (derived from ensemble mean of 17 ANT simulations, forced with historical GS forcing only). This comparison reveals that the simulated and projected anthropogenic climate change signals are consistent, whereas in all seasons the simulated GS signal is within the range of projected GS signals derived from 17 models. In the following sections we first test the significance of the observed SLP trends and then apply the consistency analysis. 

### 4.1. Are the Observed SLP Trends Derived from an Undisturbed Stationary Climate?

Figure 2 shows observed seasonal and annual area mean changes of mean sea level pressure (SLP) over the period 1975–2004. The observed trend is likely not derived from an undisturbed stationary climate in cases where the 90% uncertainty ranges (red whiskers in Figure 2) based on control variability (Section 3.3) exclude zero. There is no significant trend in the annual area mean change of SLP, but at seasonal resolution the observations indicate highly enhanced seasonality over the Mediterranean region (Figure 2).

As shown by red whiskers in Figure 2, there is less than a 5% chance (one-sided test) that observed trends in winter, summer, and autumn are due to natural (internal) variability alone. Thus, observed trends cannot be explained by natural (internal) variability derived from 6,200-year control runs; externally forced changes are detectable in all seasons, except spring.

Therefore, in the second step we investigate whether the observed trends, which are found to be inconsistent with natural (internal) variability, are consistent with what models simulate as response of SLP to anthropogenic (GS) forcing. We separate the consistency process in two parts.The first is searching for projected-observed data correspondence at the spatial-mean level by using uncentered pattern similarity indices. The second is considering this also at the pattern level, by employing centered pattern similarity indices. The results of our analysis can be interpreted as follows: the GS forcing is detectable if the pattern similarity indices are significantly greater than zero,but not detected if the indices are negative or not significantly greater than zero. Consistency of observed trend patterns with GS signal pattern is claimed in cases where the uncertainty range of regression indices, derived from control runs, does not include zero but includes unit scaling.

### 4.2. Consistency of Spatially Averaged Changes

 Here we investigate the consistency of observed trend pattern with GS signal patterns using uncentred pattern similarity statistics (1) and (3).

In winter the observed trend pattern shows a high pattern correlation with all the 17 GS signal patterns. The correlation coefficients are in the range of 0.55–0.96, and in most cases higher than 0.80 (see Table 2). The correlation coefficients between the observed and GS signal patterns are further compared with the distribution of the 90th percentile of correlation coefficients of 206 unforced trends (derived from 6,200-year control variability) with anthropogenic (GS) signal pattern. The comparison indicates that in winter all the 17 GS signal patterns yield a correlation with none of the 206 unforced trends as high as that with observed trends. Therefore the significantly (with probability of error of less than 5%) greater than zero high pattern correlation coefficients with all the 17 GS signal patterns lead us to conclude that the large-scale component of GS signal has a detectable influence in observed wintertime positive sea level pressure trend.

In spring the correlation coefficients are in the range 0.20–0.85 and with 12 of 17 models significantly (at 5%) greater than zero. A smaller pattern correlation is found in summer; here indices are in the range of 0.47–0.78 and with 11 of 17 cases greater than the 90th percentile of pattern correlations of 206 control run segments. In autumn we found negative correlation with all 17 GS signal patterns (Table 2); however, indices are out of the range of the distribution of 90th percent %tile correlation coefficients of 206 unforced trends, pointing to the fact that externally forced changes are detectable in observed patterns, which are inconsistent with GS forcing.

**Figure 3:**
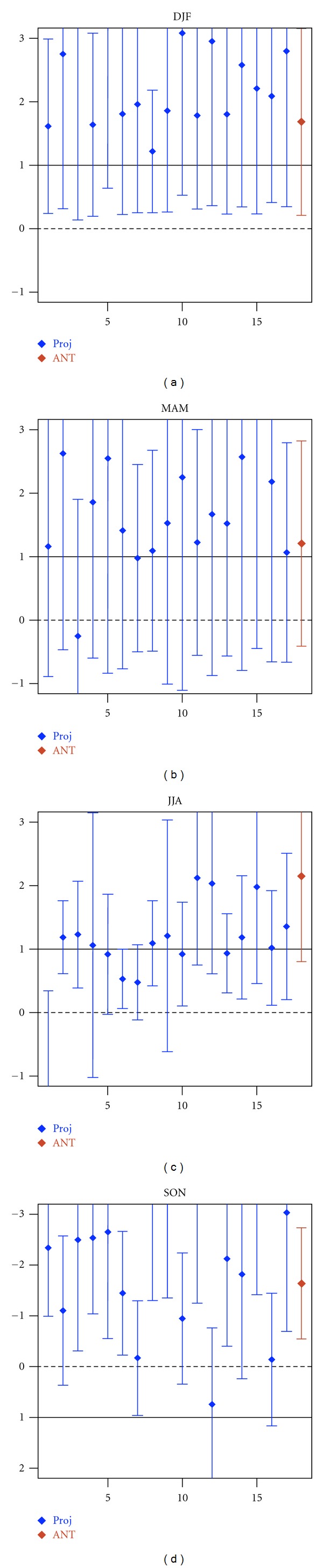
Results from consistency of spatially averaged changes. Blue bars: seasonal uncentred regression coefficients (*y* axes) of observed SLP changes onto the changes in response to GS forcing derived from 17 climate change projections (*x* axes) based on the SRES A1B scenario. Red bars: regression coefficient of observed SLP changes onto the simulated GS signal estimated from ensemble mean of 17 ANT simulations (conducted with 9 models). Whiskers show the 90% uncertainty ranges of regression coefficients derived from model-based estimates of natural (internal) variability (206 nonoverlapping 30-year segments derived from 6,200-year control runs). The solid lines mark regression indices equal to unity indicating consistency with GS forcing.

We also use regression as a pattern, similarity measure, which unlike the correlation statistics, measures the relative magnitude of observed and simulated trend patterns.  Figure 3 displays the uncentred regression coefficients of observed SLP changes into the GS signal patterns derived from the 17 model projections. In winter the uncentred regression indices are positive and significantly greater than zero (the uncertainty range of regression indices dose not include zero line) for all the 17 models, indicating that GS signal is detectable. In addition, the uncertainty range of the regression indices includes unity in all cases, demonstrating that the observed trend pattern in winter is consistent with the GS signal patterns, derived from 17 climate change projections (with the probability of error of less than 5%).

In summer the 90% uncertainty range of the regression indices does not include the zero line either, but includes unit scaling for 12 of the 17 models (blue bars in Figure 3). Therefore, we conclude that there is less than 5% chance that natural (internal) variability rather than GS signal is responsible for the observed summertime SLP changes, and the observed trend pattern in summer is consistent in 12 of the 17 GS signal patterns.

In autumn, in contrast to other seasons, the negative but significantly greater than zero regression indices (blue bars in Figure 3) indicate that external forcings are detectable in the observed trend, but other forcings rather than GS signal are responsible for the observed unusual negative SLP trends in autumn.

Overall, significant uncentred correlation and uncentered regression indices clearly indicate that the combined large-scale and small-scale component of GS signal is detectable within all the 17 models in winter and within 12 of the 17 models in summer. In addition, the GS forcing is a plausible explanation of the observed positive trend in winter and negative trend in summer (with probability of error of less than 5%).

We further investigate the robustness of the results against estimating the GS signal from ANT simulations outlined in Section 3.1. We use ensemble mean of 17 ANT simulations conducted with 9 models solely forced by historical anthropogenic forcing. Results indicates that in winter and summer the observed trends correlate significantly with the GS forcing pattern (at 5% level); correlation is 0.90 in winter and 0.78 in summer (see Table 2). When using uncentred regression indices (red bars in Figure 3), the influence of GS forcing is also detectable in winter at 5% level and in summer at 1% level. Therefore, obtained results in this section are robust against using simulated GS signal pattern.

### 4.3. Consistency of Anomaly Patterns

 Our level of confidence in attributing observed changes to anthropogenic influence will be increased if we could demonstrate that even regional features of a model-projected anthropogenic signal, and not only the area average, are consistent with the observed changes. This is where centred statistics are useful since they focus on anomalies around the area mean [19, 20]. In this section we investigate the consistency of the observed and GS anomaly patterns, when the spatial mean is removed, using centred pattern similarity indices (2) and (4).

In winter the correlations between the observed and projected GS anomaly patterns are in the range 0.53–0.88. Significant test indicates that in winter 11 of 17 GS anomaly patterns yield a correlation with none of the 206 unforced trends as high as that with observed trends (with probability of error less than 5%). However, in summer we find no correlation between observed and projected GS anomaly patterns.

**Figure 4:**
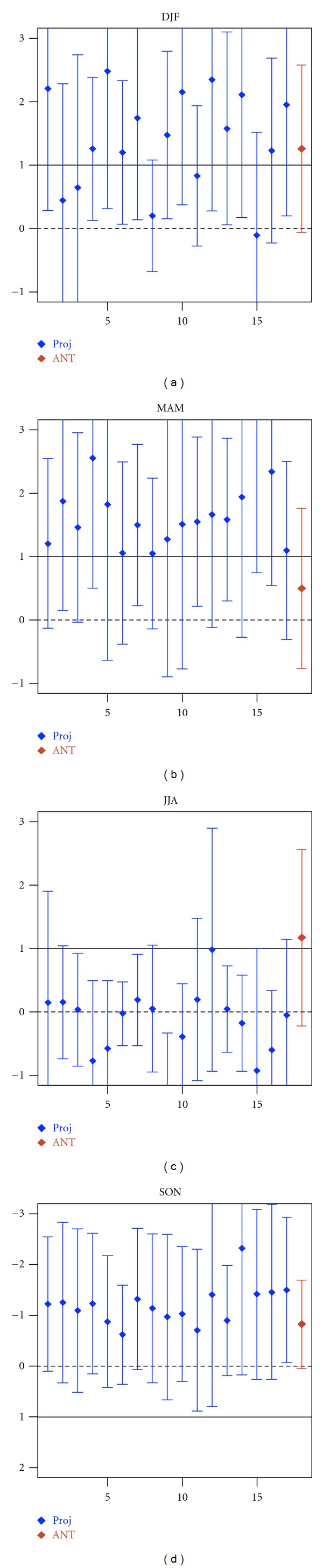
Results from consistency of anomaly patterns. Blue bars: seasonal centred regression coefficients (*y*-axes) of observed SLP changes onto the GS signal patterns estimated from 17 climate change projections (*x*-axes) based on the SRES A1B scenario. Red bars: regression coefficient of observed SLP onto the ensemble mean of 17 ANT simulations (conducted with 9 models). The bars show the 90th percentile uncertainty ranges of regression coefficients derived from model-based estimates of natural (internal) variability (206 nonoverlapping 30-year segments derived from 6200-year control runs). The solid lines mark regression indices equal to unity indicating consistency with GS forcing.

Figure 4 displays the centred regression indices and their respective uncertainty ranges. Analysis of the plots displayed in Figure 4 reveals that when subtracting the spatial mean and comparing the anomaly patterns, the small-scale component (spatial anomalies about the mean) of GS signal is detectable in winter with 11 of the 17 models. However, in summer the anomaly component of GS signal is hardly identifiable, suggesting that in summer the spatial-mean of GS signal is the dominant component.

Therefore, our consistency analysis at pattern level reveals that the small-scale component (spatial anomalies about the spatial mean) of GS signal is detectable only in winter within 11 of 17 models.

## 5. The Robustness of the Results against Observationally Based Internal Variability

 Uncertainty in estimating climate variability plays a critical role to make inferences whether GS signal is detectable in the observed changes. This uncertainty is addressed here by further using an observationally based estimation of internal variability, instead of the estimation used in previous sections based on control simulations. We estimate interval variability by resampling the observed record (available from 1850 to 2004 based on HadSLP2.0 dataset). To preserve the serial correlation present in the observations, we use a method of re-sampling called *z* moving blocks bootstrap technique [21]. It consists in randomly drawing blocks of fixed length from the measured data, rather than single years, and concatenating them. The blocks can appear several times in the surrogate series. A good proportion of the original autocorrelation is thus preserved within each block. The disadvantage of this method is that the observed record is relativity short (124 years in this study) and may be affected by the response to external forcing; the advantage is that internal variability is produced from the real world itself not from model simulations.

The block length chosen for the moving blocks bootstrapping depends on the autocorrelation of the seasonal sea level pressure time series. This autocorrelation is different for each grid box and each season. Our analysis, based on a method suggested by Wiks [21], indicates that the average block length across the Mediterranean is 3.4, 2.2, 1.8, and 2.5 years for DJF, MAM, JJA, and SON, respectively. Thus, we draw 1000 30-year time series to estimate the variability of 30-year trends in a stationary climate.

**Figure 5:**
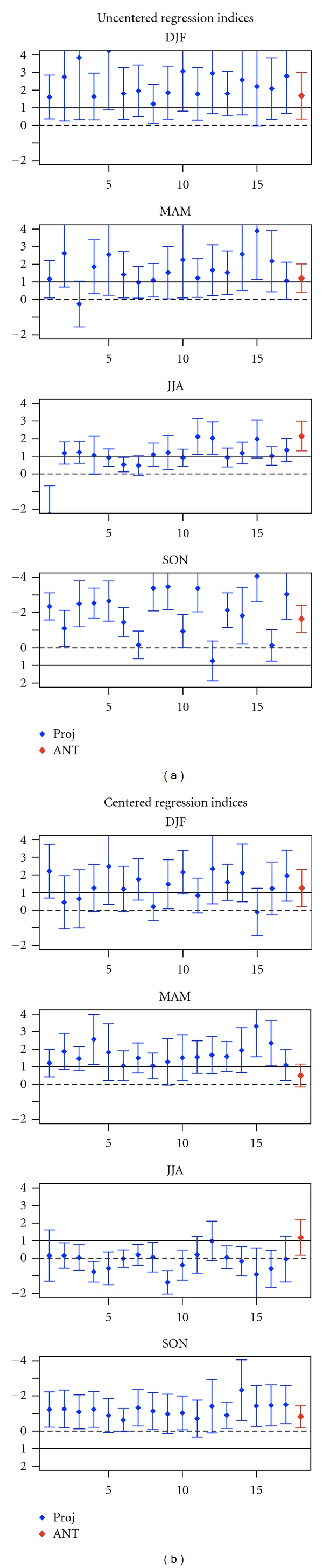
Seasonal uncentred (a) and centered (b) regression indices (*y*-axes) of observed SLP changes onto the changes in response to GS forcing derived from 17 climate change projections (*x*-axes) based on the SRES A1B scenario (blue bars) and regression coefficient of observed SLP changes onto the simulated GS signal estimated from ensemble mean of 17 ANT simulations (red bars). Whiskers show the 90% uncertainty ranges of regression coefficients derived from observationally based internal variability, estimated from block bootstrapping. The solid lines mark regression indices equal to unity indicating consistency with GS forcing.

Figure 5 displays the uncentred and centered regression indices with individual models and their 90% uncertainty ranges, derived from observationally based internal variability. Our results show that in spring the internal variability based on block bootstrapping is slightly smaller than variability based on control runs. Therefore, we are also able to detect the externally forced changes in spring (Figure 5). As shown in Figure 5 (left column), the GS signal is detectable and observed trends are consistent with GS signal patterns in 16 of the 17 models in winter, in 16 of the 17 models in spring, and in 12 of the 17 models in summer (at 5% significant level). Analysis of anomaly patterns (Figure 5, right column) reveals that the smaller-scale component of GS signal is also detectable in 8 out of the 17 models in winter and in 13 of 17 models in spring, at 5% significant level (Figure 5).

Overall, our results are the same when using observationally based internal variability derived from block bootstrapping; however, the effect of GS signal, both at spatial mean level and at pattern level, is also detectable in observed springtime SLP changes, which was not detectable using estimation of internal variability based on control runs.

## 6. The Influence of the NAO

 The Mediterranean climate is affected by the North Atlantic atmospheric circulation. The NAO is a measure of a pressure teleconnection and is defined as a large-scale alternation of atmospheric mass between regions of subtropical high pressure (the Azores high) and subpolar low pressure (the Icelandic low) in the North Atlantic. The NAO is the main feature driving the climate of western Mediterranean (see [22], and references therein), and the displacement of the Azores High, linked to NAO, leads to blocking episodes or to enhancement/weakening of the westerlies over Western Europe [23]. Ribera et al. [24] have shown that sea level pressure behavior over the Mediterranean region is dominated by QBO (quasi-periodic oscillation of the equatorial zonal wind between easterlies and westerlies in the tropical stratosphere) while NAO modulates the amplitude of sea level pressure. It is now recognized that models may underestimate the natural variability of this atmospheric circulation.Therefore, in this section we explore the consequences of subtracting in the observations that part of the sea level pressure variability that can be attributable to the NAO.

For the NAO we use a station-based NAO index [25].The fingerprint of the NAO is defined as the fraction of the variability in SLP time series, which covaries with the NAO index.Thus we define the fingerprint of the NAO as the slope of the regression of SLP time series on the NAO index for each grid box separately.The NAO signal is removed from observed trends by subtracting the trend in the NAO index times the NAO signal from the trend in the observations.

**Figure 6:**
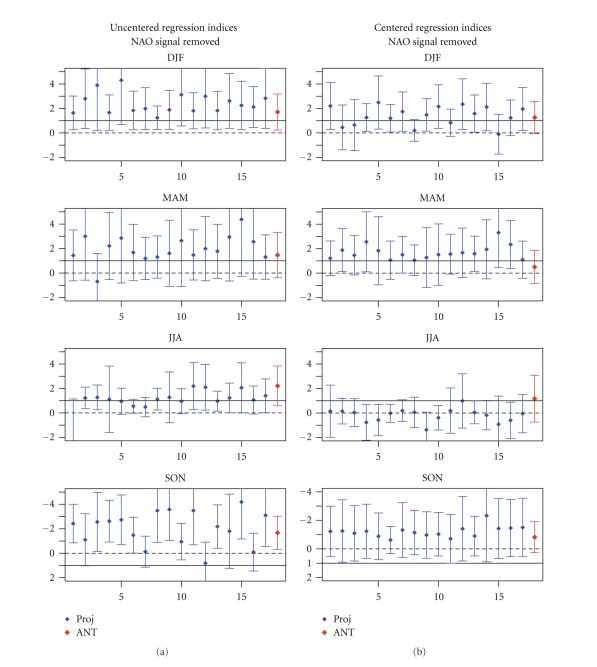
Same as Figures 3 and 4, but after removing the NAO signal.

The obtained results after removing the fingerprint of NAO is shown are Figure 6. Results show that the NAO hardly affects the SLP trends, and the removal of the NAO signal does not considerably change the observed trends.Therefore the detection of the influence of anthropogenic (GS) forcing in winter and summer is robust against the removal of the NAO fingerprint.

## 7. Analysis of the Frequency of Extreme SLP Days

 In this section we investigate the role of GS forcing in the frequencies of extreme low and high mean SLP days over the Mediterranean area. The frequencies in the extremes of low/high mean SLP days were computed using daily mean sea level pressure reconstructions for the period 1974–2003 [13]. The extreme values of mean SLP were established considering as threshold values those of the 10th percentile, for the lowest values and of the 90th percentile for the highest one. Days with SLP less than the 10th percentile value are defined as low SLP days and those greater than the 90th percentile are defined as high SLP days. The remaining days are considered as normal mean SLP days [10, 11].

The same procedure as that used for mean SLP is applied for estimating the frequency of extreme SLP days in response to GS forcing. One time-slice experiment, conducted with the INGV climate model, is used to estimate anthropogenic climate change signal. The scenario run with the INGV model covers the period until 2070, and therefore, the GS signal is defined as the difference between 2041–2070 and the reference climatology (1961–1990). The resulting signal is scaled to change per decade. Control simulations (1,200 year) are used to estimate the variability of the extreme mean SLP values in a stationary climate.

**Figure 7:**
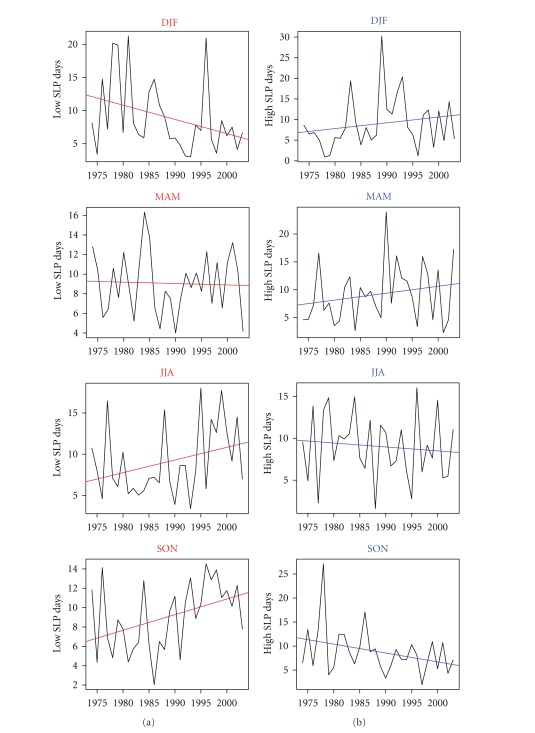
(b) Temporal evolutions of the frequencies of HIGH SLP days. (a) Temporal evolutions of the frequencies of low SLP days, in DJF (winter), MAM (spring), JJA (summer) and SON (autumn) over the time period from 1974 to 2003. The corresponding best-fit linear trend lines are included.

**Figure 8:**
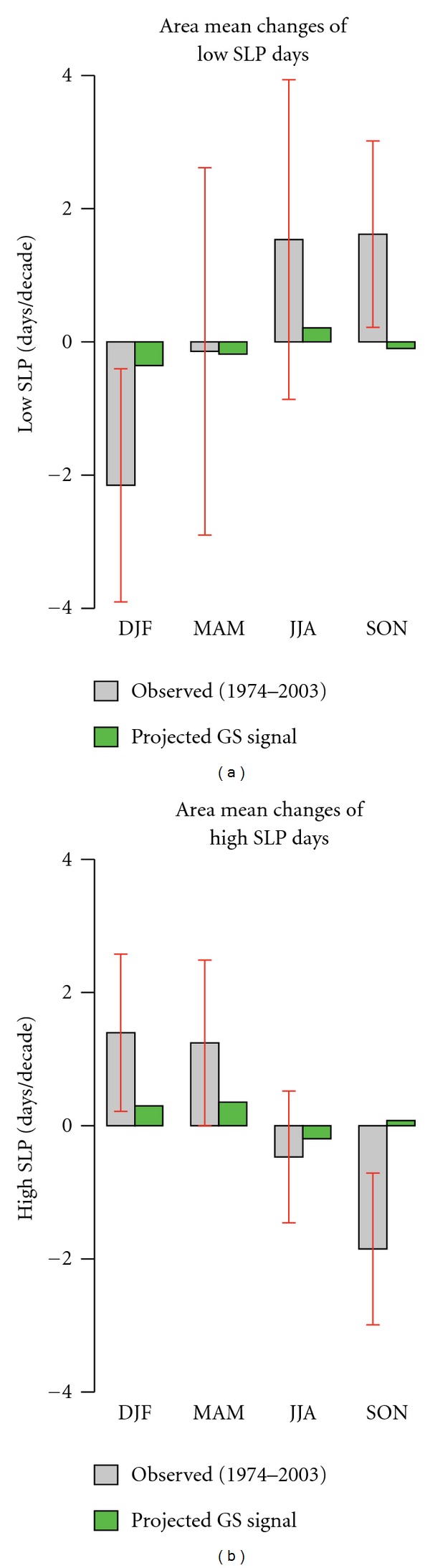
(a) Observed seasonal area mean change (grey bars) in the frequency of low SLP days (days with mean SLP less than the 10th percentile). (b) Observed seasonal area mean change (grey bars) in the frequency of high SLP days (days with mean SLP greater than the 90th percentile) over the period from 1974 to 2003 in comparison with GS signal pattern (GS) according to A1B scenario derived from INGV model (green bars). The vertical axes show trends in the number of low (high) days per decade. The red whiskers indicate the 90% uncertainty range of observed trends derived from natural (internal) variability estimated from 1,200-year control simulations (40 nonoverlapping 30 year segments).

Figure 7 shows the temporal evolution of the frequencies of low and high SLP days in each season over the period 1974–2003. The trend lines included in the plots point to the existence of trends in all seasons during the study period.  Figure 8 displays seasonal area mean changes of low SLP days (days with SLP less than the 10th percentile) and high SLP days (days with SLP higher than the 90th percentile) over the period from 1974 to 2003 in comparison with GS signal pattern derived from INGN model. The red whiskers denote the 90% uncertainty range of observed trends estimated from 1,200-year control simulations (40 nonoverlapping 30-year segments).

In winter (DJF) the trends are found to be positive for the frequency of the high SLP days and negative for those of low SLP days (Figures 7 and 8). In spring also the observed trends in the frequency of high SLP days are positive indicating, consequently, a reduction in observed winter and springtime precipitation amount over the Mediterranean region. In contrast to winter and spring, in summer and autumn the observed trends are positive for the case of low SLP days, whereas in the case of high SLP days the trends are negative, indicating an increase in cyclonic activities and a decrease in anticyclonic activities. After 1980, in summer and autumn the trends in the frequency of low (high) SLP days are seen to increase (decrease) with +5 (−0.3) and +4 (−2.5) days per decade, respectively.

As shown by red whiskers in Figure 8 observed trends in winter and autumn are inconsistent with simulated natural (internal) variability, and thus externally forced changes are detectable at 5% level. Therefor, we estimate a less than 5% probability that the changes in the frequency of low/high SLP days as strong as that observed in winter and autumn could occur in unforced coupled models.

In the following we assess the probability that GS forcing is a plausible explanation for the observed changes in the frequency of low/high SLP days over the Mediterranean region, which can not be explained by natural (internal) variability. For this purpose we compare the observed seasonal trends (1974–2003) with those estimated by the INGV model as the response of extreme SLP to GS forcing.

**Figure 9:**
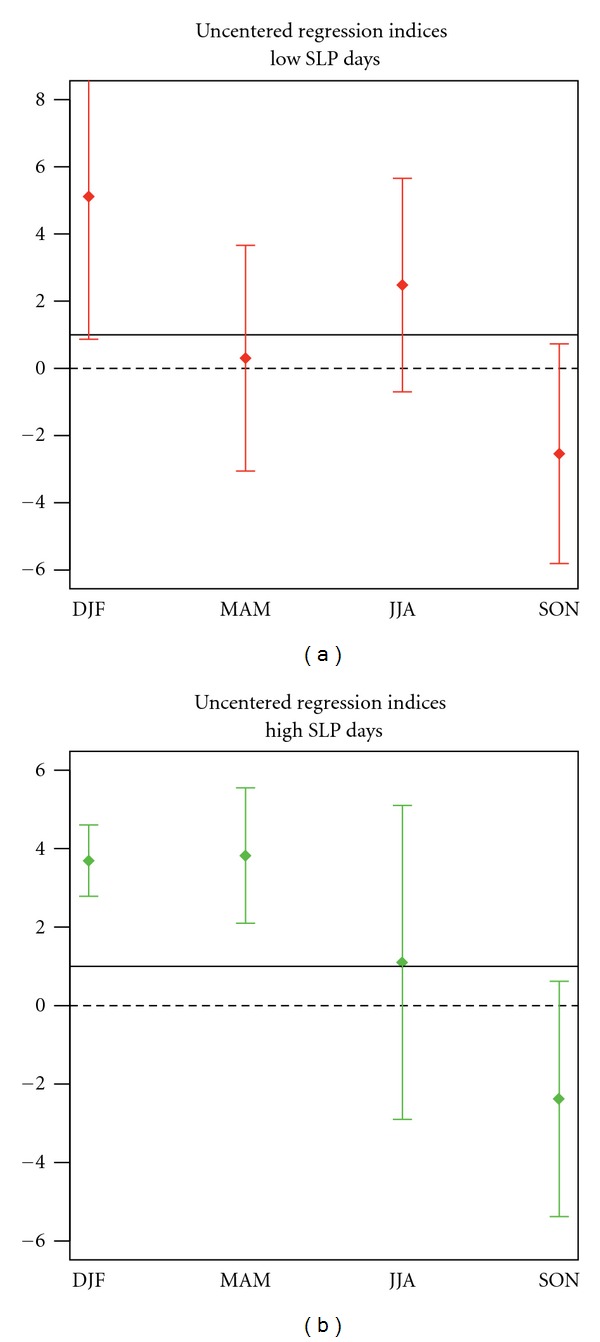
Uncentred regression coefficients of observed changes in the frequency of low/high SLP days against changes projected by INGV model in response to GS forcing. The whiskers indicate the 90% uncertainty range of regression indices derived from control variability (1,200-year control simulations). Solid lines indicate regression indices equal to unity, indicating consistency with GS forcing.

In winter the pattern of observed extreme low (high) SLP days shows a high correlation with the pattern of GS signal correlation 0.88 (0.80) and significantly greater than zero (with less than 5% risk).  Figure 9 displays uncentred regression coefficients of observed changes in the frequency of low/high SLP days against changes projected by INGV model in response to GS forcing. As shown in Figure 9, in winter the large-scale component of the GS signal is detectable in the observed pattern of the frequency of low and high SLP days. However observed SLP changes are inconsistent with projected response to GS forcing, and INGV model strongly underestimates the increasing (decreasing) tendency in the frequency of observed high (low) SLP days (the centered regression indices are greater than 3). In autumn, the negative regression indices (see Figure 9) point to the present of an external forcing, which is not GS forcing. Therefore, we conclude that the increasing (decreasing) tendency of the frequency of the low (high) SLP days in autumn, which goes along with more precipitation over the Mediterranean region, might have rather a natural than anthropogenic (GS) origin.

## 8. Summary and Conclusions

 In this study, we try to assess the influence of GS forcing in observed mean SLP trends, using a large set of climate change projections. We investigate whether the observed mean SLP trends over the period 1975–2004 are similar to what models projected and simulated as response of SLP to anthropogenic forcing. We separate the consistency process in two parts.The first is searching for projected-observed data correspondence at the spatial-mean level by using uncentered pattern similarity indices. The second is considering this also at the pattern level, by employing centered pattern similarity indices. The statistical significance of this similarity is estimated in two ways: by using 6,200-year control runs (control simulations with all forcings held constant) and by estimating the internal variations of the SLP trends by resampling the observations. We further examine the role of GS forcing in the frequencies of the extremes of low/high mean SLP days. To determine whether the days is a low or high mean SLP day, the statistical percentile thresholds are computed based on the daily data for the period 1974–2003 during the seasons. Control simulations (1,200-year) are used to estimate the variability of the extreme mean SLP values in a stationary climate.

Between 1975 and 2004 period observed mean SLP has increased in winter and spring, while observed record suggests a decrease in mean SLP in summer and autumn over the Mediterranean region. Results indicate that no single sample of 206 segments, derived from control variability, yields a trend of SLP as strong as that observed during the period 1975–2004, in all seasons except spring (with probability of error of less than 5%). Therefor we conclude that observed SLP trends can not be explained by natural(internal) variability alone and externally forced changes are significantly (at 5% level) detectable in all seasons, except spring.

The significantly greater than zero high uncentred correlation coefficients, along with significant uncentred regression indices, clearly indicate that the large-scale component (spatial mean) of the GS signal is detectable in the observed mean SLP trends in winter and summer and that the observed trends are significantly consistent with all 17 GS signal patterns in winter and in 12 of the 17 GS signal patterns in summer (at 5% level). Our consistency analysis of the anomaly patterns reveals that the smaller-scale (spatial anomalies about the spatial mean) component of the GS signal is only detectable within 9 of the 17 models in winter. However, in summer we failed to find consistency between the observed and projected spatial anomaly patterns, indicating that in summer the spatial mean of GS signal is the dominant component. These results are robust to using simulated GS signal, derived from ensemble mean of 17 ANT simulations (conducted with 9 models). Our results are also robust against using observationally based internal variability. We further investigate the influence of the NAO and find that obtained results in this study are robust against the removal of the NAO fingerprint.

In addition, we find that in winter the observed trends (1974–2003) in the frequency of low/high SLP days are not consistent with internal variability, and the response to external forcing is detected (at 5% level). Obtained results show that the large-scale component of GS signal is detectable (at 5% level) in winter. However, there are discrepancies in the magnitude of changes. The projected GS signal strongly underestimates the observed negative (positive) trends in the number of low (high) SLP days in winter and thus consequently may underestimate the anticyclonic activities in winter, which may have already had a significant effect on agriculture in the Mediterranean region. These results support the results of our analysis of precipitation trends [8], which demonstrates that whereas the precipitation response to GS is detectable in wintertime, the observed negative trends in precipitation are several times larger than expected changes due to GS forcing.

In winter (DJF), the decreasing (increasing) tendency of the frequency of the low (high) SLP days suggests a consistency between the extreme SLP and its associated rainfall activities. In 1983, 1989, 1992, and 1993 Mediterranean region has experienced extreme high pressure systems during the 21, 32, 18, and 22 days per 90 days, respectively, and strong decrease in the amount of precipitation [8]. Interestingly, two major volcanic eruptions in the last two centuries occurred during this time period: one in 1982 and 1991. These eruptions injected large amount of ashes and gases in the form of aerosols into the atmosphere and could impact the global climate (about 15 million tons of sulfur dioxide). Therefore, the large volcanic eruptions might have triggered, to some extent, the extreme high frequency of high SLP days in 1992, 1993, and 1983 in winter and ultimately reducing the rainfall activity over the region. Thus, the observed extreme daily high pressure systems and consequently reduction in wintertime precipitation amount over the Mediterranean region might be partly natural.

While the detection of an anthropogenic influence on observed mean and extreme SLP trends is robust in winter, there are inconsistencies in autumn, where analysis points to the presence of an external forcing, which is not GS forcing. In autumn, observed changes show a large negative trend in the mean SLP, as well as increase (decrease) in low (high) SLP days, these trends are inconsistent with internal variability and also inconsistent with GS signal patterns. Therefore, we conclude that the increasing (decreasing) tendency of the frequency of the low (high) SLP days in autumn, which goes along with more precipitation over the Mediterranean region [8], might have a natural rather than an anthropogenic (GS) origin. This kind of inconsistency must be reconciled if we want to employ the scenarios as realistic anticipations of possible future regional climate change.

## Figures and Tables

**Table 1:** The seventeen coupled ocean-atmosphere models and the number of twentieth century runs with each individual model. The control integrations (runs) used in this study to estimate natural (internal) variability.

	Models (number of runs)	Number of years used form control integrations
1	BCCR-BCM2-0 (1)	250 (8 30-year segments)
2	CCCMA-CGCM3-1 (5)	1000 (33 30-year segments)
3	CCCMA-CGCM3-1-t63 (1)	350 (11 30-year segments)
4	CNRM-CM3 (1)	500 (16 30-year segments)
5	CSIRO-MK3-0 (1)	350 (11 30-year segments)
6	GFDL-CM2-0 (1)	500 (16 30-year segments)
7	GFDL-CM2-1 (1)	500 (16 30-year segments)
8	GISS-AOM (2)	250 (8 30-year segments)
9	INGV-ECHAM4 (1)	100 (3 30-year segments)
10	INMCM3-0 (1)	350 (11 30-year segments)
11	MIROC3-2-HIRES (1)	100 (3 30-year segments)
12	MIROC3-2-MEDRES (3)	500 (16 30-year segments)
13	MIUB-ECHO-G (3)	350 (11 30-year segments)
14	MPI-ECHAM5 (4)	500 (16 30-year segments)
15	MRI-CGCM2-3-2A (5)	350 (11 30-year segments)
16	NCAR-CCSM3-0 (7)	230 (7 30-year segments)
17	UKMO-HADGEM1 (1)	160 (5 30-year segments)

		Total: 206 non-overlapping 30-year segments

**Table 2:** Seasonal uncentred pattern correlation coefficients of mean sea level pressure for 30-year trends from 1974 to 2005, compared to the trend of 17 anthropogenic (GS) signal patterns and ensemble mean of 17 ANT simulations conducted with 9 models, derived from the CMIP3 multimodel data set. The indices significantly greater than zero at 5% level are labelled with an asterisk.

	Models	DJF	MAM	JJA	SON
1	BCCR-BCM2-0	0.92*	0.70*	0.40	−0.95*
2	CCCMA-CGCM3-1	0.67*	0.63*	0.50*	−0.16
3	CCCMA-CGCM3-1-T63	0.55*	0.20	0.61*	−0.40*
4	CNRM-CM3	0.89*	0.85*	0.21	−0.94*
5	CSIRO-MK3-0	0.86*	0.55	0.67*	−0.69*
6	GFDL-CM2-0	0.87*	0.68*	0.30	−0.40*
7	GFDL-CM2-1	0.93*	0.78*	0.19	−0.20
8	GISS-AOM	0.73*	0.76*	0.39*	−0.74*
9	INGV-ECHAM4	0.91*	0.32	0.39	−0.77*
10	INMCM3-0	0.90*	0.62	0.67	−0.19
11	MIROC3-2-HIRES	0.81*	0.80*	0.64*	−0.71*
12	MIROC3-2-MEDRES	0.90*	0.78*	0.78*	0.10
13	MIUB-ECHO-G	0.96*	0.84*	0.47*	−0.59*
14	MPI-ECHAM5	0.90*	0.69*	0.65*	−0.18
15	MRI-CGCM2-3-2A	0.57*	0.81*	0.59*	−0.81*
16	NCAR-CCSM3-0	0.89*	0.84*	0.67*	−0.02
17	UKMO-HADGEM1	0.91*	0.73*	0.73*	−0.50

	GS signal (17 ANT Simulations)	0.90*	0.37*	0.78*	−0.51*

## References

[1] Gillett NP, Zwiers FW, Weaver AJ, Stott PA (2003). Detection of human influence on sea-level pressure. *Nature*.

[2] Gillett NP, Allan RJ, Ansell TJ (2005). Detection of external influence on sea level pressure with a multi-model ensemble. *Geophysical Research Letters*.

[3] Shindell DT, Schmidt GA, Miller RL, Rind D (2001). Northern Hemisphere winter climate response to greenhouse gas, ozone, solar, and volcanic forcing. *Journal of Geophysical Research D*.

[4] Marshall GJ, Stott PA, Turner J, Connolley WM, King JC, Lachlan-Cope TA (2004). Causes of exceptional atmospheric circulation changes in the Southern Hemisphere. *Geophysical Research Letters*.

[5] Wang XL, Swail VR, Zwiers FW, Zhang X, Feng Y (2009). Detection of external influence on trends of atmospheric storminess and northern oceans wave heights. *Climate Dynamics*.

[6] Gillett NP, Stott PA (2009). Attribution of anthropogenic influence on seasonal sea level pressure. *Geophysical Research Letters*.

[7] Barkhordarian A, Bhend J, von Storch H Consistency of observed near surface temperature trends with climate change projections over the Mediterranean region.

[8] Barkhordarian A, von Storch H, Bhend J The expectation of future precipitation change over the Mediterranean region is different from what we observe.

[9] Meehl GA, Covey C, Delworth T (2007). The WCRP CMIP3 multimodel dataset—a new era in climatic change research. *Bulletin of the American Meteorological Society*.

[10] Labajo JL, Martín Q, Labajo AL, Piorno A, Ortega M, Morales C (2008). Recent trends in the frequencies of extreme values of daily maximum atmospheric pressure at ground level in the central zone of the Iberian Peninsula. *International Journal of Climatology*.

[11] Labajo JL, Labajo AL, Piorno A, Martín Q, Ortega MT, Morales C (2009). Analysis of the behavior of the extreme values of minimum daily atmospheric pressure at ground level over the Spanish Central Plateau. *Atmosfera*.

[12] Allan R, Ansell T (2006). A new globally complete monthly historical gridded mean sea level pressure dataset (HadSLP2): 1850–2004. *Journal of Climate*.

[13] Ansell TJ, Jones PD, Allan RJ (2006). Daily mean sea level pressure reconstructions for the European-North Atlantic region for the period 1850–2003. *Journal of Climate*.

[14] Gualdi S, Scoccimarro E, Navarra A (2008). Changes in tropical cyclone activity due to global warming: results from a high-resolution coupled general circulation model. *Journal of Climate*.

[15] Bhend J, von Storch H (2008). Consistency of observed winter precipitation trends in northern Europe with regional climate change projections. *Climate Dynamics*.

[16] Bhend J, von Storch H (2009). Is greenhouse gas forcing a plausible explanation for the observed warming in the Baltic Sea catchment area?. *Boreal Environment Research*.

[17] Gillett NP, Zwiers FW, Weaver AJ, Hegerl GC, Allen MR, Stott PA (2002). Detecting anthropogenic influence with a multi-model ensemble. *Geophysical Research Letters*.

[18] Hegerl G, Zwiers F (2011). Use of models in detection and attribution of climate change. *Wiley Interdisciplinary Reviews: Climate Change*.

[19] Santer BD, Wigley TML, Jones PD (1993). Correlation methods in fingerprint detection studies. *Climate Dynamics*.

[20] Santer BD, Taylor KE, Wigley TML, Penner JE, Jones PD, Cubasch U (1995). Towards the detection and attribution of an anthropogenic effect on climate. *Climate Dynamics*.

[21] Wilks DS (1997). Resampling hypothesis tests for autocorrelated fields. *Journal of Climate*.

[22] Xoplaki E, Gonzalez-Rouco JF, Luterbacher J, Wanner H (2004). Wet season Mediterranean precipitation variability: influence of large-scale dynamics and trends. *Climate Dynamics*.

[23] Rogers JC (1997). North Atlantic storm track variability and its association to the North Atlantic oscillation and climate variability of Northern Europe. *Journal of Climate*.

[24] Ribera P, Garcia R, Diaz HF, Gimeno L, Hernandez E (2000). Trends and interannual oscillations in the main sea-level surface pressure patterns over the Mediterranean, 1955–1990. *Geophysical Research Letters*.

[25] Osborn TJ (2006). Recent variations in the winter North Atlantice Oscillation. *Weather*.

